# Supplementary material for: Causes and consequences of sex-chromosome turnovers in Diptera
Source: Evol Lett. 2026 Mar 12;10(3):253–68. doi: 10.1093/evlett/qrag003 (PMC13231274; doi:10.1093/evlett/qrag003)
Supplement: qrag003_Supplemental_Files [file qrag003_supplemental_files.zip › Supplementary_Datasets.zip!/Supplementary_Datasets/Dataset_2_Graphical_representation_outgroups/Tephritidae_Anastrepha_ludens_chromosomes.pdf]

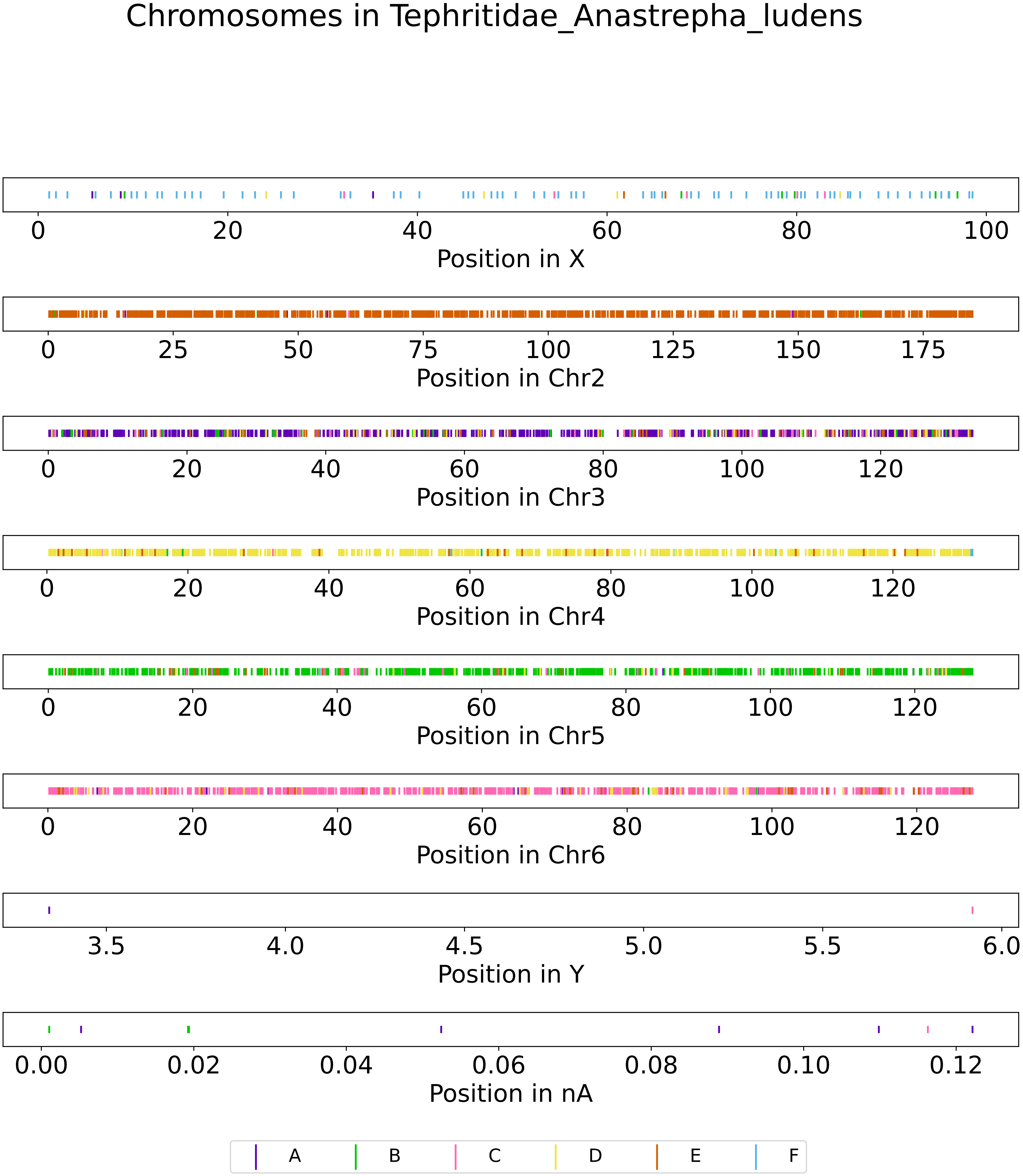
Chromosomes in Tephritidae_Anastrepha_ludens
0
20
40
60
80
100
Position in X
0
25
50
75
100
125
150
175
Position in Chr2
0
20
40
60
80
100
120
Position in Chr3
0
20
40
60
80
100
120
Position in Chr4
0
20
40
60
80
100
120
Position in Chr5
0
20
40
60
80
100
120
Position in Chr6
3.5
4.0
4.5
5.0
5.5
6.0
Position in Y
0.00
0.02
0.04
0.06
0.08
0.10
0.12
Position in nA
A
B
C
D
E
F
